# Repeated Exposure to High-THC *Cannabis* Smoke during Gestation Alters Sex Ratio, Behavior, and Amygdala Gene Expression of Sprague Dawley Rat Offspring

**DOI:** 10.1523/ENEURO.0100-23.2023

**Published:** 2023-11-27

**Authors:** Thaisa M. Sandini, Timothy J. Onofrychuk, Andrew J. Roebuck, S. Austin Hammond, Daniel Udenze, Shahina Hayat, Melissa A. Herdzik, Dan L. McElroy, Spencer N. Orvold, Quentin Greba, Robert B. Laprairie, John G. Howland

**Affiliations:** 1 Department of Anatomy, Physiology, and Pharmacology, University of Saskatchewan, Saskatoon, Saskatchewan S7N 5E5, Canada; 2 School of Liberal Arts, Yukon University, Whitehorse, Yukon Territory Y1A 5K4, Canada; 3 Global Institute for Food Security, Saskatoon, Saskatchewan S7N 4L8, Canada; 4 Next Generation Sequencing Facility, Saskatoon, Saskatchewan S7N 5E5, Canada; 5 Deparment of Oncology, University of Saskatchewan, Saskatoon, Saskatchewan S7N 5E5, Canada; 6 College of Pharmacy and Nutrition, University of Saskatchewan, Saskatoon, Saskatchewan S7N 5E5, Canada

**Keywords:** behavior, cannabinoid, development, prenatal

## Abstract

Because of the legalization of *Cannabis* in many jurisdictions and the trend of increasing Δ^9^-tetrahydrocannabinol (THC) content in *Cannabis* products, an urgent need exists to understand the impact of *Cannabis* use during pregnancy on fetal neurodevelopment and behavior. To this end, we exposed female Sprague Dawley rats to *Cannabis* smoke daily from gestational day 6 to 20 or room air. Maternal reproductive parameters, offspring behavior, and gene expression in the offspring amygdala were assessed. Body temperature was decreased in dams following smoke exposure and more fecal boli were observed in the chambers before and after smoke exposure in dams exposed to smoke. Maternal weight gain, food intake, gestational length, litter number, and litter weight were not altered by exposure to *Cannabis* smoke. A significant increase in the male-to-female ratio was noted in the *Cannabis*-exposed litters. In adulthood, male and female *Cannabis* smoke-exposed offspring explored the inner zone of an open field significantly less than control offspring. Gestational *Cannabis* smoke exposure did not affect behavior on the elevated plus maze test or social interaction test in the offspring. *Cannabis* offspring were better at visual pairwise discrimination and reversal learning tasks conducted in touchscreen-equipped operant conditioning chambers. Analysis of gene expression in the adult amygdala using RNA sequencing revealed subtle changes in genes related to development, cellular function, and nervous system disease in a subset of the male offspring. These results demonstrate that repeated exposure to high-THC *Cannabis* smoke during gestation alters maternal physiological parameters, sex ratio, and anxiety-like behaviors in the adulthood offspring.

## Significance Statement

*Cannabis* use by pregnant women has increased alongside increased tetrahydrocannabinol (THC) content in recent years. As smoking *Cannabis* is the most common method of use, we used a validated model of *Cannabis* smoke exposure to repeatedly expose pregnant rats to combusted high-THC *Cannabis* smoke. Our results show alterations in litter sex ratio, anxiety-like behavior, and decision-making in the offspring that may relate to subtle changes in expression of amygdala genes related to development, cellular function, and nervous system disease. Thus, we believe this gestational *Cannabis* exposure model may be useful in delineating long-term effects on the offspring.

## Introduction

Given the recent legalization and decriminalization of *Cannabis* in many jurisdictions around the world, there is an urgent need to better understand its effects in a variety of populations, especially in pregnant women. Indeed, in the United States, 4–7% of pregnant women report using *Cannabis* (Ko et al., 2015; Volkow et al., 2019), and 16–38% of those self-report daily use (Ko et al., 2015; Pike et al., 2021; Metz et al., 2022; Satti et al., 2022). *Cannabis* use among pregnant and lactating women is largely motivated by its purported antiemetic properties, and for stress and anxiety relief (Westfall et al., 2006; Skelton et al., 2020; Satti et al., 2022). Population-based studies have also shown long-term effects of maternal *Cannabis* use during pregnancy on offspring development, emotionality, cognition, and brain function (Fried and Smith, 2001; Smith et al., 2006; Higuera-Matas et al., 2015; Smith et al., 2016; Crume et al., 2018; Sharapova et al., 2018; Bara et al., 2021; Moore et al., 2022b). Cannabinoids readily cross the placenta barrier and modulate endogenous cannabinoid signaling, which is involved in developmental processes including uterine implantation, neurogenesis, neurite outgrowth, synapse development, and axon targeting (Wu et al., 2011; Higuera-Matas et al., 2015; Alpár et al., 2016; Richardson et al., 2016; Grant et al., 2018; Hurd et al., 2019; Scheyer et al., 2019; Bara et al., 2021). However, research on the effects of *Cannabis* on human neonatal outcomes, prenatal development, and long-term behavior are still limited. In addition, the availability of *Cannabis* strains with higher Δ^9^-tetrahydrocannabinol (THC) content has steadily increased in recent years (Smart et al., 2017) raising concerns about higher doses of cannabinoids reaching the developing fetus.

Preclinical models are essential to evaluate the effects of gestational *Cannabis* exposure in a more controlled manner, and the information obtained allows for better understanding of the underlying neurobiological mechanisms and long-term behavioral consequences of exposure (Schneider, 2009; Scheyer et al., 2019; Bara et al., 2021). In rats, gestational exposure to injected cannabinoids (i.e., THC or the synthetic cannabinoid type 1 receptor agonist WIN55,212-2) compromises fetal growth (Natale et al., 2020) and normal brain development, leading to cognitive deficits in the offspring (Ferraro et al., 2009). Gestational treatment with WIN55,212-2 (0.5 mg/kg, s.c.) from gestational day 5 (GD5) to GD20, reduces social interaction (SI), ablates endocannabinoid-mediated long-term depression, and heightens excitability of prefrontal cortex pyramidal neurons in male, but not female, offspring (Bara et al., 2018). Additional studies support these findings, showing the male, but not female, offspring of pregnant dams exposed to THC (2 mg/kg, s.c.; GD5-20) exhibit a hyperdopaminergic phenotype and increased sensitivity to challenge with THC during adolescence (Frau et al., 2019; Traccis et al., 2021). However, most gestational exposure studies have used maternal injections of cannabinoids, which do not reflect the pharmacokinetics of smoked or ingested cannabinoids or the potential entourage of compounds consumed with whole-plant products (Russo, 2011; Moore et al., 2022a). Thus, maternal injections have poor face validity when compared with common administration methods used by humans, which include inhalation of *Cannabis* smoke or vaporized extracts and oral consumption. In addition, differences in pharmacokinetics because of the route of administration significantly influence the amount of fetal exposure (Baglot et al., 2021, 2022). In this context, the exposure of female rats to vaporized, high-THC *Cannabis* extracts before and during pregnancy altered measures related to anxiety and behavioral flexibility of both male and female offspring (Weimar et al., 2020). Thus, use of *Cannabis* exposure paradigms that mimic human consumption patterns are an important goal of recent, ongoing, and future work.

In the present experiments, we chose to assess the effects of repeatedly exposing pregnant rats to the smoke of commercially available dried, high-THC *Cannabis* flowers from GD6 to GD20. We chose flowers high in THC (∼20%), given the THC content in strains of *Cannabis* readily available today. The exposure protocol was developed for use in adult rats and has been shown to result in measurable THC concentrations in plasma, as well as behavioral changes immediately following smoke exposure (Barnard et al., 2022; Roebuck et al., 2022). Recent pharmacokinetic data from the plasma of pregnant rat dams confirm that repeated exposures to high-THC *Cannabis* smoke results in THC levels of ∼20–25 ng/ml 30 min following the initiation of exposure (Black et al., 2023). During and after *Cannabis* smoke exposure, we quantified an array of maternal and offspring parameters related to acute effects of smoke exposure on the pregnant dams, litter health, and behavior of the male and female offspring in early adulthood. We also assayed the lasting effects of *Cannabis* smoke exposure on gene expression in the amygdala of the offspring using RNA sequencing (RNA-Seq), given reports of altered anxiety-like behavior (Trezza et al., 2008; Weimar et al., 2020) and amygdala gene expression (Spano et al., 2007) in previous studies of gestational *Cannabis* and THC exposure. Given the literature reviewed above, we hypothesized that *Cannabis* smoke exposure would alter neonatal development and long-term behavioral phenotypes of male and female adulthood offspring.

## Materials and Methods

### Animals

Sexually naive female (*n* = 22) and male (*n* = 12) Sprague Dawley rats (70 d old; Charles River) were pair housed by sex in a temperature-controlled (21°C) and light-controlled (12 h light/dark cycle) vivarium managed by the Lab Animal Safety Unit at the University of Saskatchewan. Water and food were available *ad libitum* in their cages. All procedures were performed during the light phase (7:00 A.M. to 7:00 P.M.) and conducted with the approval of the local Animal Research Ethics Board.

After a week of acclimatization to the vivarium, female rats were handled for 3 d (3 min/rat) and were then habituated to the smoke chamber for 20 min/d for 4 d before being bred in the vivarium. The habituation was performed before breeding to reduce stress before the early implantation period (GD5). Rats were habituated to the smoke chambers with the pumps off for 2 d, and then with the pumps on for the following 2 d. During the habituation and maternal smoke exposure, dams were placed individually in cages (16 × 25 × 13 (h) cm) in the smoke chambers, and either one or two cages were exposed in each smoke chamber.

### Breeding

**Figure 1.**
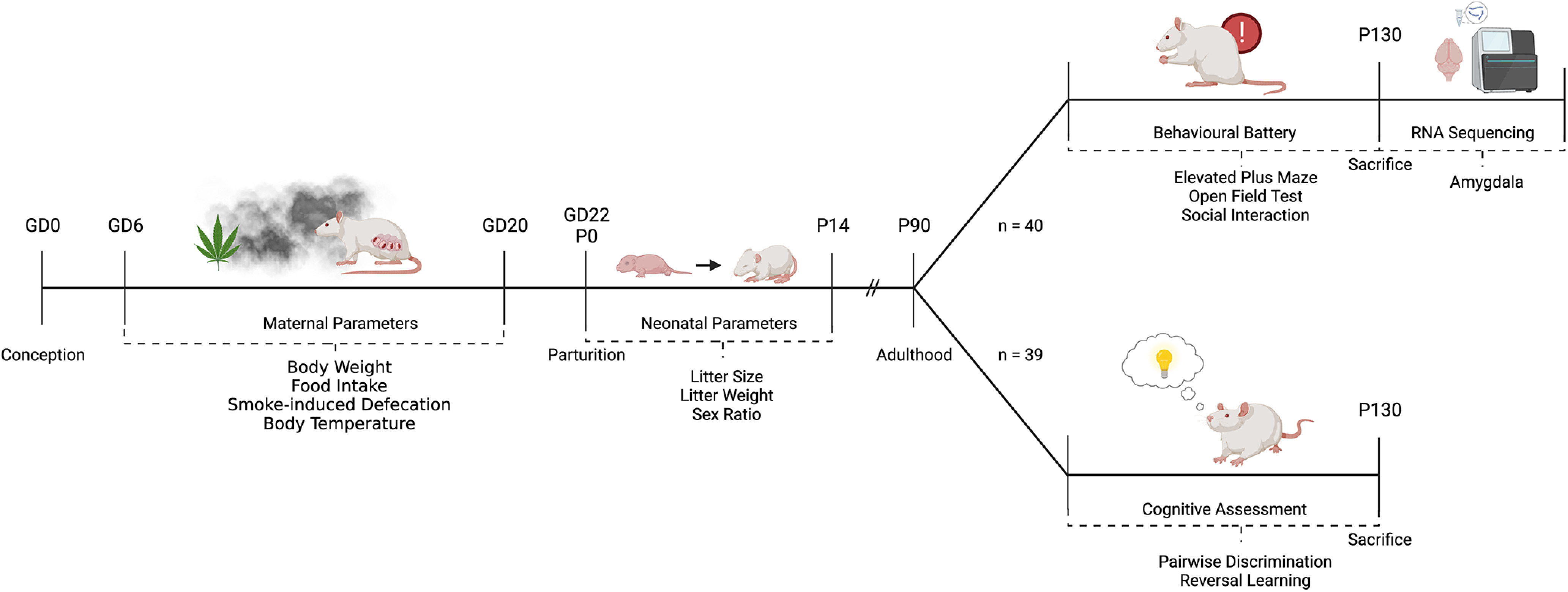
Schematic depicting the flow of dams and their litters through this experiment. Rats were bred in our facility and exposed to either room air or high-THC *Cannabis* smoke from GD6 to GD20. Litters were assessed for basic parameters during the neonatal period and then left to develop until adulthood (P90). Offspring were then split into 2 cohorts for behavioral and cognitive assessment. Offspring used in the behavioral battery were killed, and RNA sequencing was performed on tissue from their amygdala. The figure was created with BioRender.com.

Complete details regarding the breeding protocol have been published previously (Sandini et al., 2020). One day before breeding began, male rats were split into individual cages. On the day of breeding, two female rats (pair housed) were put in the male cage overnight. The following morning (8:00 A.M.), cells were collected from the vagina of each rat with a sterile P200 pipette tip filled with 50–60 μl of sterile physiological saline. Pregnancies were confirmed by the presence of spermatozoa visualized at the light microscope, and this day was considered GD0. After pregnancy was confirmed, rats were singly housed and followed the timeline depicted in Figure 1. Smoke exposure was initiated on GD6 given the risk of spontaneous abortions with earlier administration (Navarrete et al., 2020) and previous cannabinoid administration protocols in rodents (Bara et al., 2018; Natale et al., 2020; Sarikahya et al., 2023). All animals were weighed every 3 d to monitor general health and pregnancy.

### Cannabis

The *Cannabis* strain used in our experiments is Mohawk, described by the retailer as an indica-dominant strain with 19.51% THC, <0.07% cannabidiol (CBD; https://www.leafly.ca/brands/aphria/products/aphria-mohawk-rockstar). All *Cannabis* used in this study was purchased from Aphria, was sourced from the same lot (lot #6216), and was shipped to our facility in December 2019 and January 2020. Each day, *Cannabis* was freshly prepared by shredding the full flower in a standard coffee grinder (∼5 s) and weighing it into 200 mg increments. This amount was chosen based on previous work examining the effects of *Cannabis* smoke of the same strain in rats (Barnard et al., 2022; Roebuck et al., 2022).

### *Cannabis* smoke exposure system

*Cannabis* smoke exposure was conducted using a validated four-chamber inhalation system commercially available from La Jolla Alcohol Research, which has been used in previous studies (Barnard et al., 2022; Roebuck et al., 2022). Before each session, *Cannabis* was packed into a ceramic bowl fixed to a metal heating coil that could be heated to combust the product. The coil was sealed with a glass lid and rubber O-ring, and the entire assembly could be connected to the atomizer and inhalation system. The inhalation system was composed of four identical chambers. Each chamber is airtight and constructed from clear Plexiglas measuring ∼33 cm (height) × 30.5 cm (width) × 51 cm (length) with an internal volume of ∼50 L. Except during smoke exposure, room air was pumped through the chambers at a flow rate of 10–12 L/min. Air was filtered and exhausted into the facility ventilation system through a fumehood.

On GD6, dams were put into the chambers as described above, and after 5 min of acclimatization and equalization of the pressure in all the chambers, the smoke session started. *Cannabis* was combusted over a period of 1 min. To ensure complete combustion, ignition occurred over 5 s and was repeated three times with a delay of 15 s between each light. Following combustion, the pumps were stopped for 1 min to allow for exposure. After exposure, pumps were restarted, and the venting process continued for 13 min. The venting was not immediate, and continued exposure of a gradually decreasing amount of *Cannabis* smoke occurred during this time. After venting was complete, dams were removed and immediately returned to their home cage. Air-control dams were exposed to the same procedure, except that no *Cannabis* was combusted.

### Treatment, maternal, and neonatal parameters

**Figure 2.**
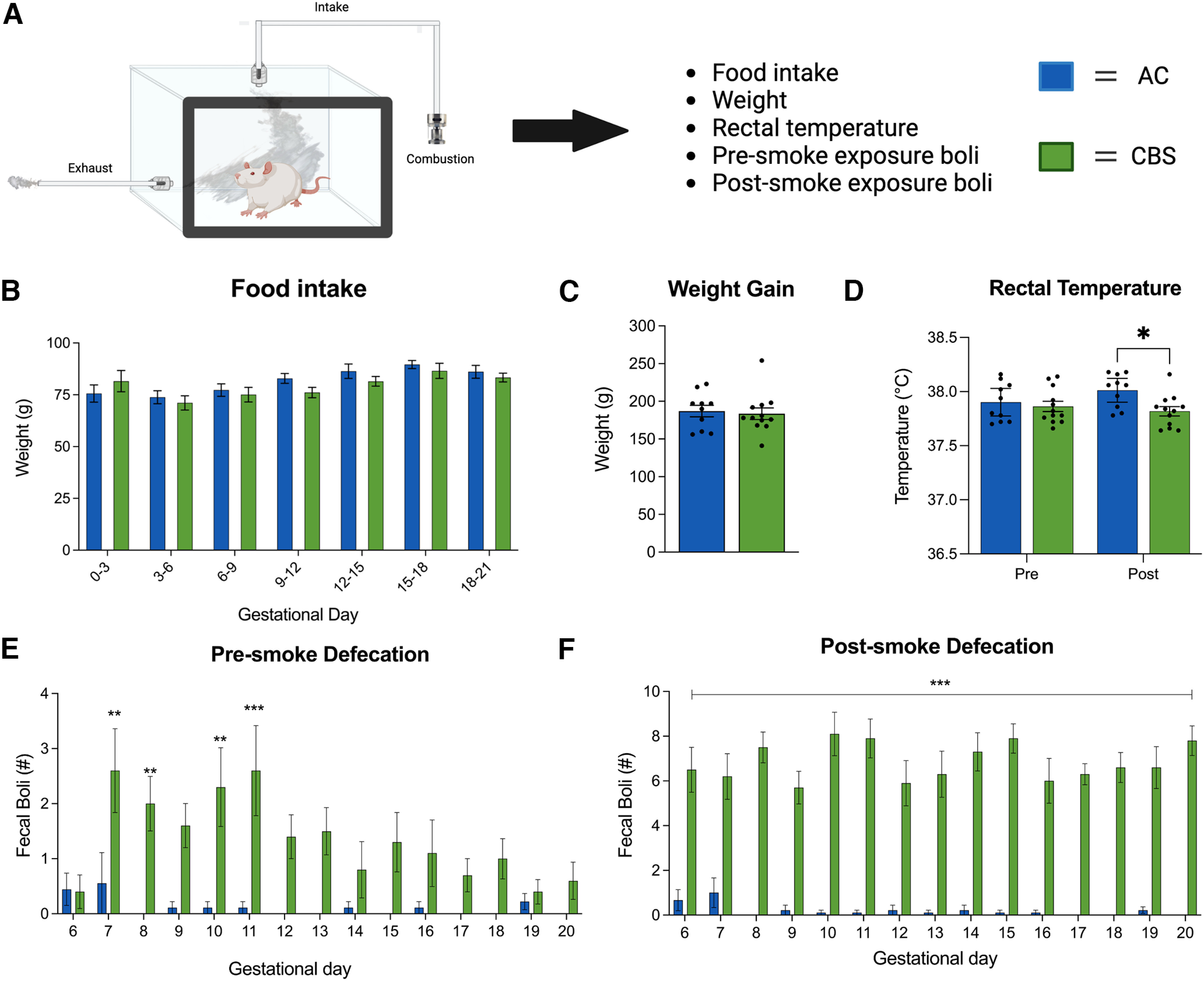
Effects of smoke exposure on the pregnant dams. ***A***, Pregnant dams were exposed to either room air or high-THC-containing *Cannabis* smoke from GD6 to GD20. A series of measurements were taken during this phase. ***B***, ***C***, *Cannabis* smoke exposure did not affect maternal food intake (***B***) or weight gain (***C***). ***D***, Exposure to smoke was associated with a significant decrease in rectal temperature, which was driven by a reduction in temperature post-treatment of the dams treated with high-THC *Cannabis* smoke. ***E***, Analyses of defecation in the smoke chambers revealed that dams treated with smoke showed increased defecation during the presmoke period for several days following the first smoke exposure. Critically, the groups did not differ before the first smoke administration (GD6). ***F***, Dams exposed to smoke consistently defecated more than air controls after the smoke was administered (postsmoke defecation). Asterisks denote significant changes between treatment groups: *p < 0.05, **p < 0.01, ***p < 0.001. AC, Air control; CBS, *Cannabis* smoke. The figure was created with BioRender.com. See Extended Data Table 2-1 for all statistics.

Pregnant rats were split into the following two groups: (1) 200 mg of *Cannabis* combusted and delivered as smoke (*n* = 12); and (2) room air control (*n* = 10). Exposures occurred once a day for 15 min from GD6 to GD20 (Figs. 1, 2). Food intake and body weight were measured every 3 d. Rectal temperatures were also taken before and immediately following exposure every 3 d. Furthermore, the number of fecal boli was measured before and after smoke exposure every day. After the 5 min acclimatization period, the experimenter quickly counted the boli through the transparent cages before initiating smoke exposure. After the smoke exposure and evacuation periods, the rats were removed from the smoke chambers and the total boli were counted. Presmoke counts were subtracted from total counts to calculate postsmoke defecation counts. After the last exposure on GD20, the rats were not handled before giving birth on GD21 to GD22. The day of birth was designated postnatal day 0 (P0). On P1, litter size, sex ratio, and live birth index were assessed, and the litters were culled to a maximum of 12 pups. When possible, an equal number of males and females were kept. On P7 and P14, litters were weighed again. Because of the COVID 19 pandemic, use of the vivarium was severely restricted when offspring entered their second postnatal month in March/April 2020. As a result, litters were culled after weaning to 77 pups for testing in the behavioral battery after P90 (Fig. 1).

10.1523/ENEURO.0100-23.2023.tab2-1 Table 2-1 Additional statistics by figure in the main manuscript. Download Table 2-1, DOCX file.

### Behavioral testing of the offspring

To test our hypothesis that gestational *Cannabis* smoke exposure causes lasting effects on the behavioral profile of offspring into adulthood, we conducted tests of locomotion, anxiety-like behavior, social behavior, and behavioral flexibility. Testing began at approximately P90 and was completed by P130 (adulthood). Offspring were divided into the following two groups: one group (*n* = 40; smoke-exposed male, *n* = 11; control male, *n* = 9; smoke-exposed female, *n* = 11; control female, *n* = 9) was first tested in the open field test (OF test), elevated plus maze test (EPM), and SI test (Fig. 1). A second group (*n* = 37; smoke-exposed male, *n* = 10; control male, *n* = 7; smoke-exposed female, *n* = 11; control female, *n* = 9) was tested in the touchscreen-based pairwise discrimination (PD) and reversal learning (RL) tasks (Fig. 1). A maximum of one pup of each sex per litter was saved and used in the behavioral battery. Testing occurred during the light phase (12:00 P.M. to 6:00 P.M.) for the OF, EPM, and SI tests, and male rats were tested before females. For PD/RL, male rats were trained during the morning (8:00 A.M. to 11:30 A.M.), and female rats during the afternoon (1:00 P.M. to 5:00 P.M.). Ethanol (40%) was used to clean all behavior testing equipment between rats. Before behavioral tests, rats were handled for a minimum of 3 min/d for 3 consecutive days. Handling included exposure to investigators and emphasized picking up and moving rats until the motions could be conducted with ease, as well as habituation to travel by cart between the animal housing and behavior testing rooms.

#### OF

The testing apparatus consisted of a circular arena (wall dimensions: diameter, 150 cm; height, 45 cm) made of industrial plastic painted black (see Fig. 4*A*). Rats were brought into the testing room and placed individually in the arena for 15 min. Distance traveled (in meters) and time spent (in seconds) in the inner area of the arena (100-cm-diameter circle centered in the middle) was analyzed using Noldus Ethovision XT (version 6) software.

#### Elevated plus maze

The EPM apparatus had two open arms and two closed arms of equal size (50 × 10 cm) with 40-cm-high walls (see Fig. 5*A*). The EPM was elevated 60 cm above the floor. Rats were placed individually in the central area facing an open arm and were allowed 5 min of free exploration. The number of entries and amount of time in open and closed arms were recorded for each rat. Rats had to place all four paws in an arm for an entry or time to be counted. Additionally, rearing, grooming, and head-dipping frequencies were evaluated by a researcher blind to treatment.

#### Social interaction

The OF apparatus was used to evaluate social interaction (see Fig. 6*A*). Both test and stranger rats freely explored the circular arena separately for a 10 min habituation period 24 h before testing, as previously described (Marks et al., 2019). During testing, a test rat and a stranger rat of the same treatment and sex were allowed to freely explore the arena for 10 min at the same time. On test day, all test rats were marked on their back with a black marker. Stranger rats were left unmarked. Behavior was scored from videos using stopwatches by a researcher blind to the treatment of the rats. Latency for the first approach (in seconds) and 20 cm proximity of the test rat were evaluated. The proximity measure included behaviors such as sniffing, nosing, chasing, and passing the stranger rat.

#### PD and RL tasks

Discrimination learning and behavioral flexibility were assessed by serial completion of touchscreen-based PD and RL tasks. Seven touchscreen-equipped operant conditioning chambers (Bussey-Saksida Touch System, Lafayette Instrument) equipped with a trapezoidal inner chamber (30.5 × 24.1 × 8.25 cm), food reward delivery system (45 mg; Dustless Precision Pellets, Rodent Purified, BioServ), overhead camera for live behavioral monitoring, and a touchscreen monitor were used (see Fig. 7*A*). For all training procedures, a black plastic mask and response shelf was placed in front of the monitor. The mask covered the entirety of the display, except for two identical rectangular cutouts located in the upper half of the screen where visual stimuli presentation would occur. System functionality checks and cleaning procedures were conducted daily (Barnard et al., 2022).

**Table 1:** Mean (±SEM) response latencies (in seconds) for the correct and incorrect trials, as well as reward collection, during the touchscreen testing

	PD		RL	
	AC	CBS	AC	CBS
Correct	5.18 ± 0.8	4.16 ± 0.4	5.45 ± 0.7	4.40 ± 0.4
Incorrect	6.65 ± 1.2	4.86 ± 0.6	5.09 ± 0.7	4.49 ± 0.5
Reward	2.05 ± 0.4	1.46 ± 0.1	1.49 ± 0.1	1.45 ± 0.1

No significant differences were observed between the treatment groups for these measures (statistics shown in Extended Data Table 2-1). AC, air control; CBS, *Cannabis* smoke.

Training procedures were conducted as per manufacturer instructions and previous work (Lins et al., 2018; Roebuck et al., 2022). Before training, male and female adult offspring were restricted to 85% free-feeding body weight to motivate reward-seeking behavior. Through a series of habituation, pretraining, and training steps, rats were shaped to develop a strong association between visual stimulus selection and a food reward, to initiate stimulus presentation, and to associate incorrection selection with illumination of a bright overhead light. In short, animals were habituated to experimenters, the route of transport, and the testing apparatus over 3 consecutive days. Next, four stepwise pretraining stages—initial touch, must touch, must initiate, punish incorrect—were completed to develop appropriate stimulus–reward associations and behavioral strategies. Sessions within each stage were 1 h in duration and restricted to a maximum of 100 selection trials, where advancement between stages was awarded based on established performance criteria. Roebuck et al. (2022) provide an in-depth explanation of all habituation and pretraining stages that were used in this study.

Upon completion of pretraining stages, animals advanced to PD, a task measuring visual discrimination learning. Within each selection trial, two distinct black and white images were simultaneously displayed on the touchscreen immediately following trial initiation. One image was always correct, regardless of its spatial location, while the other image was always incorrect. Correct responses were reinforced by a food reward, and incorrect responses were punished by illumination of a bright overhead light and a 5 s delay. Incorrect responses were also directly followed by a correction trial. Here, the location of each image was identical to the previously incorrect selection trial, and advancement to a new selection trial could only be achieved by a correct selection. Criterion for advancement in PD was defined as >85% accuracy for 100 selection trials within 1 h on two consecutive training sessions. The final phase progression was RL, a test of behavioral flexibility. RL was identical to PD, except the correct image is reversed; said differently, selection of the previously incorrect image was rewarded, and selection of the previously correct image resulted in a 5 s delay and a subsequent correction trial. Criterion for completion was defined as >85% accuracy across 100 selection trials within 1 h on two consecutive training sessions. Two male rats (one from each treatment group) failed to complete the criterion for completion of RL, and, as a result, their data were not included in the analysis.

### Statistical analysis—behavioral data

Data were analyzed using GraphPad Prism version 8.0.1 software. All figures show means with the error bars showing the SEM. Before analysis, data were tested for normality of distribution using the Shapiro–Wilk test. Unpaired *t* test was used to analyze gestational length, litter size and weight, and male/female ratio. Two-way ANOVA (followed by Bonferroni’s multiple-comparisons test) with factors of Treatment (air control vs *Cannabis* smoke) and Time (gestational days) were used to evaluated maternal gain weight, food intake, body temperature, and preboli and postboli smoke exposure. For the offspring behavioral testing, two-way ANOVA (followed by Bonferroni’s multiple-comparisons test) with factors of Treatment (air control and *Cannabis* smoke) and Sex (male, female) were used when data were parametric. Nonparametric Mann–Whitney tests were used when data were nonparametric. *p* Values of ≤0.05 were considered significant.

### Gene expression in the offspring amygdala

#### Sample collection

After behavioral testing, the offspring from the OF, EPM, and SI cohort (*n* = 40) were anesthetized with isoflurane and decapitated. Amygdala tissue samples were quickly dissected, and flash frozen in liquid nitrogen for gene expression analysis.

#### RNA collection and library preparation

Total RNA was extracted from the amygdala of adult rats using the MagMAX mirVana Total RNA Isolation Kit (Thermo Fisher Scientific). RNA quality was assessed using the Qubit RNA BR Assay (Thermo Fisher Scientific) and RNA Screentape (Agilent). Sequencing libraries were constructed from 300 ng of RNA per sample using the TruSeq Stranded mRNA Library Prep Kit (Illumina).

#### RNA sequencing

Sequencing libraries were evaluated using the Qubit dsDNA BR Assay (Thermo Fisher Scientific) and a D1000 Screentape (Agilent). The barcoded libraries were pooled equimolar and 75 bp paired-end reads were generated on a NextSeq 550 instrument (Illumina).

#### Data processing

The reads were extracted from each run using bcl2fastq (version 2.19.0.316). Sequencing adapters and low-quality bases were trimmed using fastp (Chen et al., 2018) with default settings. The reads were aligned to the *Rattus norvegicus* reference genome (GCA_000001895.4) using subjunc (from subread version 2.0.1) and gene-level expression quantified using htseq-count from the HTSeq framework (version 0.11.3) with default settings except for “–nonunique all, stranded=reverse” (Anders et al., 2015).

#### Differential gene expression

Differential expression between exposed and unexposed rats was assessed using Autonomics (version 1.3.0) in RStudio (version 4.1.2). Using Autonomics pipeline, RNA expression counts were log transformed and fitted to the “∼ 0 + subgroup” model using the limma package. Significant genes for the contrast between rats exposed to *Cannabis* smoke during gestion (Treated) and unexposed (Control) rats were extracted using a Benjamini–Hochberg adjusted *p* value [false discovery rate (FDR) < 0.05] and absolute fold change (FC) of 1.5. Signaling pathways, biological and disease functions, and regulatory network interactions associated with differentially expressed genes (DEGs) were identified using Ingenuity Pathway Analysis (IPA; QIAGEN). Genes with absolute fold change >1.5 were included in the analysis, and molecules and relationships to be considered were filtered for “tissues/cell lines=amygdala, brain, CNS cell lines, or fibroblast cell lines.” Raw data related to these analyses have been posted on the NCBI repository under BioProject Accession no. PRJNA886744.

## Results

### Gestational *Cannabis* smoke exposure alters maternal responsivity to the smoke chambers and offspring sex ratio

Pregnant rats were exposed to *Cannabis* smoke once daily from GD6 to GD20, and a variety of measures were taken from dams and offspring at birth (Figs. 1, 2*A*). Gestational *Cannabis* exposure did not affect maternal food intake (Fig. 2*B*) or weight gain (Fig. 2*C*) during pregnancy (Extended Data Table 2-1, statistics). However, rectal temperature was significantly affected by treatment, with rats in the *Cannabis* smoke group showing significantly lower temperatures after treatment (Fig. 2*D*; significant Treatment-by-Phase interaction: *F*_(1,20)_ = 10.88, *p* = 0.004, *post hoc p* < 0.05). This effect did not differ significantly over the course of repeated treatments (Treatment-by-Phase-by-Day interaction *F*_(4,80)_ = 0.054, *p* = 0.99; Extended Data Table 2-1, all statistics).

When compared with air controls, rats exposed to *Cannabis* smoke from GD6 to GD20 defecated more in the smoke chambers during presmoke and postsmoke periods. Analysis of presmoke boli showed significant main effects of Treatment (Fig. 2*E*; *F*_(1,255)_ = 71.47, *p* < 0.0001) and GD (*F*_(14,255)_ = 1.94, *p* = 0.02), and a significant interaction (*F*_(14,255)_ = 1.72, *p* = 0.05). *Post hoc* analyses indicated that smoke-exposed dams produced significantly more boli on GD7, GD8, GD10, and GD11 than air controls. During the postsmoke period, *Cannabis*-exposed dams produced significantly more boli than air controls regardless of GD (Fig. 2*F*; main effect of Treatment: *F*_(1,255)_ = 755.0, *p* < 0.0001; with no effect of GD (*F*_(14,255)_ = 0.69, *p* = 0.78) or an interaction (*F*_(14,255)_ = 1.03, *p* = 0.42).

**Figure 3.**
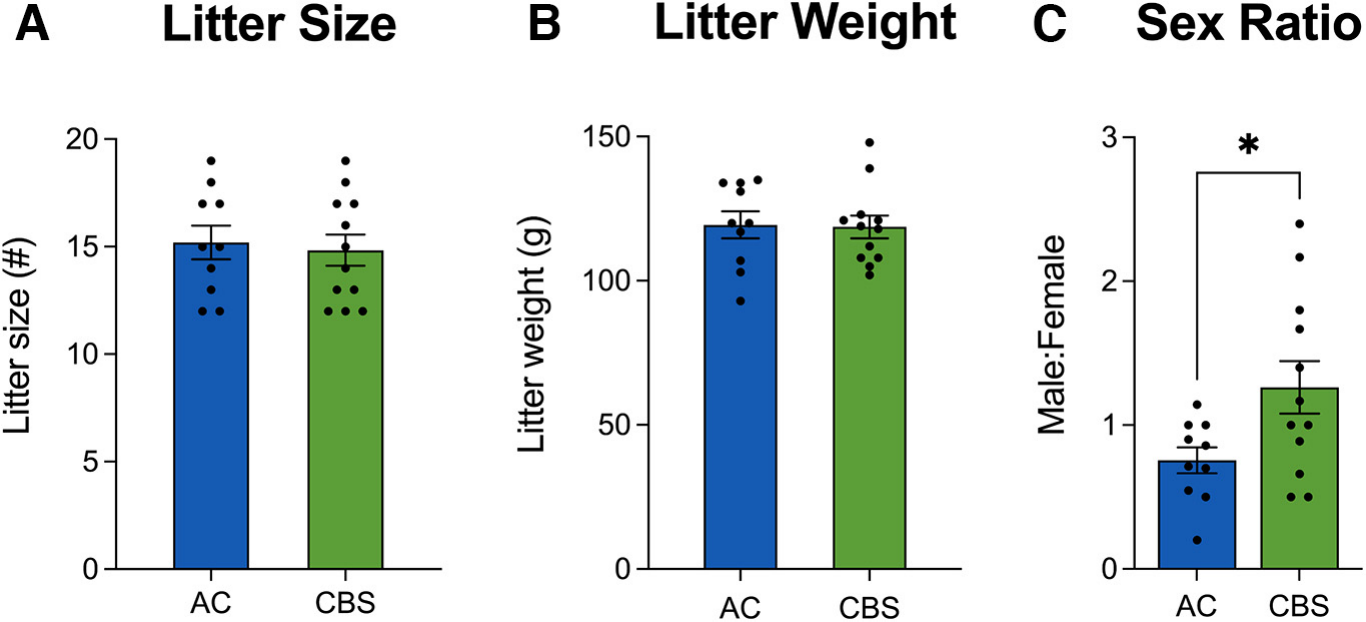
***A***, ***B***, High-THC smoke exposure did not alter the litter size (***A***) or litter weight (***B***), when compared with air controls. ***C***, High-THC smoke exposure led to a significant increase in the male/female pup ratio. Asterisks denote significant changes between treatment groups: *p < 0.05. AC, Air control; CBS, *Cannabis* smoke. See Extended Data Table 2-1 for all statistics.

Dams were allowed to give birth naturally and litters were assessed on P1. No differences were observed in the number of total pups born (Fig. 3*A*) or total litter weight on P1 (Fig. 3*B*). However, a significant increase in the male/female ratio was observed in litters exposed to *Cannabis* smoke (*t*_(20)_ = 2.34, *p* = 0.03). All litters were culled to 12 pups per dam. The average litter weight on P7 (air control = 230.6 ± 5.2; *Cannabis* exposed = 231.6 ± 5.9) and P14 (air control = 406.2 ± 8.0; *Cannabis* exposed = 404.7 ± 10.7) were not significantly altered by *Cannabis* smoke exposure (Extended Data Table 2-1, statistics).

### Gestational *Cannabis* smoke exposure alters activity in the OF test, but not in EPM or SI test

**Figure 4.**
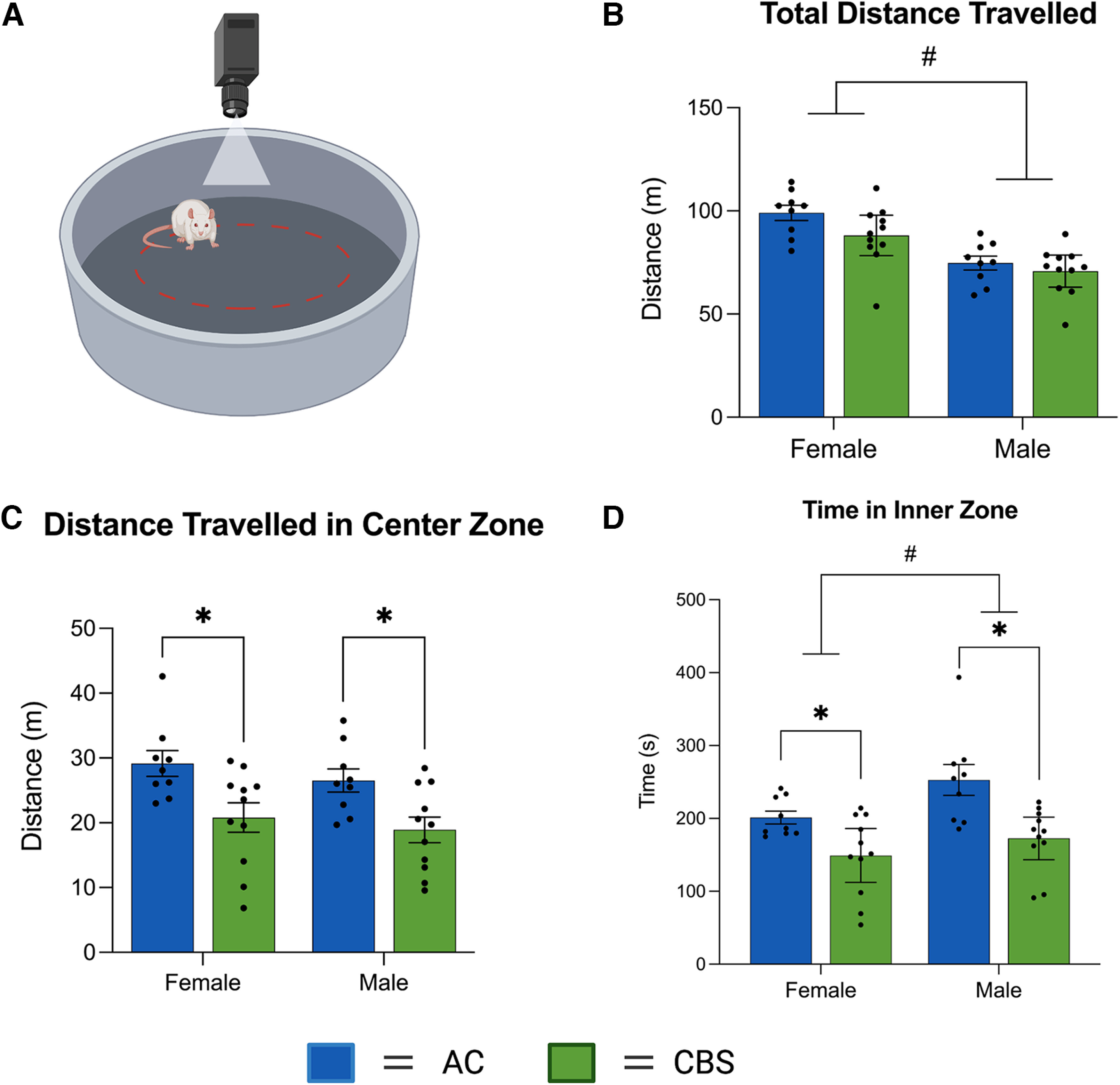
***A***, Exploratory and anxiety-like behaviors were assessed in the open field test. ***B***, Female offspring, regardless of treatment, displayed significantly more locomotor activity during the test. ***C***, ***D***, The offspring of dams treated with high-THC *Cannabis* smoke traveled less distance (***C***) and spent less time (***D***) in the inner region of the open field. Female offspring, regardless of treatment, also spent less time in the inner zone of the open field. Asterisks denote significant changes between treatment groups (*p* < 0.05). #Significant sex differences (*p* < 0.05). The figure was created with BioRender.com. See Extended Data Table 2-1 for all statistics.

Distance traveled (in meters) within the entire circular arena (Fig. 4*A*) was analyzed to examine general locomotor activity in the OF (Fig. 4*B*). Gestational *Cannabis* smoke exposure did not alter the total distance traveled (Fig. 3*A*; *F*_(1,36)_ = 3.69, *p* = 0.06); however, there was a significant effect of Sex (*F*_(1,36)_ = 29.37, *p* = 0.0001) without an interaction (*F*_(1,36)_ = 0.83, *p* = 0.36). Inspection of the data revealed that female offspring traveled farther than their male siblings, regardless of treatment. To assess anxiety-like behavior, we quantified distance traveled (Fig. 4*C*) and time spent (Fig. 4*D*) in the inner zone of the OF. Analyses of these data showed that the *Cannabis* smoke-exposed offspring traveled less and spent less time in the inner zone [main effect of Treatment for distance (*F*_(1,36)_ = 15.02, *p* < 0.001) and time (*F*_(1,36)_ = 17.80, *p* < 0.001)] of the OF. There was also a main effect of Sex for time in the inner zone (*F*_(1,36)_ = 5.74, *p* = 0.02), with females spending less time in the inner zone than males, regardless of treatment (Fig. 4*D*). All other main effects and interactions were not significant (Extended Data Table 2-1, statistics).

**Figure 5.**
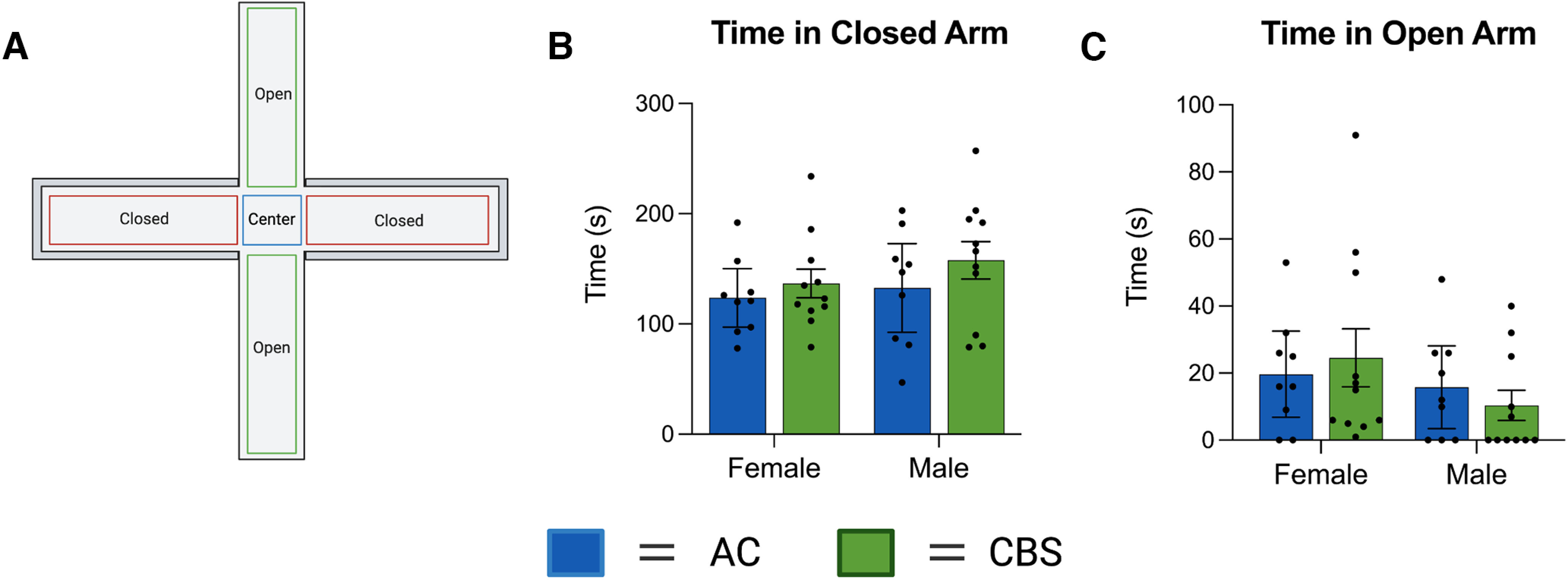
***A–C***, Assessment of the offspring in the elevated plus maze (***A***) revealed no significant differences in the time in the closed arms (***B***) or open arms (***C***). The figure was created with BioRender.com. See Extended Data Table 2-1 for all statistics.

The EPM test (Fig. 5*A*) was used as another index to measure anxiety-like behavior following gestational *Cannabis* smoke exposure in the same offspring as were used for the OFT. Two-way ANOVAs indicated no main effects of Treatment, Sex, or interactions when time spent exploring the closed and open arms (Fig. 5) was considered (Extended Data Table 2-1, statistics). Also, no differences were noted in the number of open and closed arm entries, rears, time spent grooming, or head dips were analyzed (all *p* values > 0.05; data not shown).

**Figure 6.**
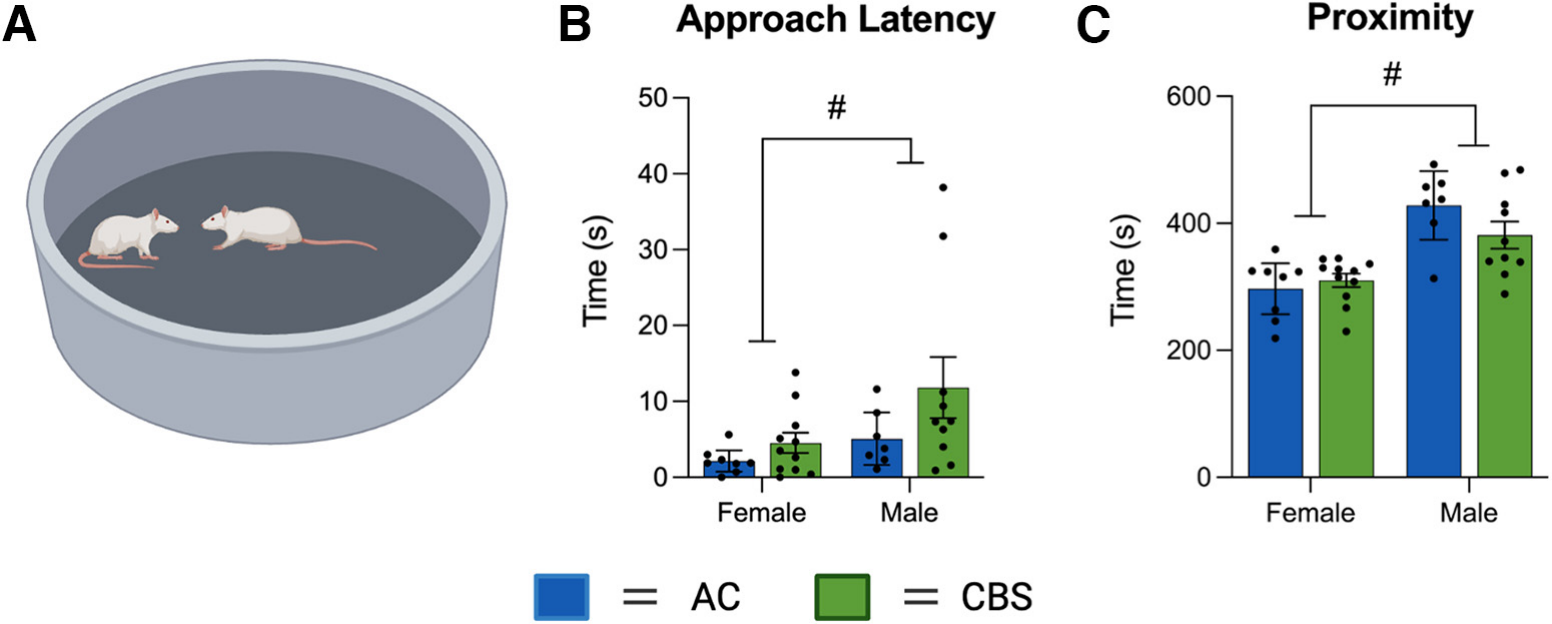
***A***, Offspring social interaction behaviors were not significantly affected by maternal *Cannabis* exposure. ***B***, ***C***, Male offspring approached (***B***) and interacted with (***C***) 20 cm more than female offspring, regardless of treatment. #Significant sex differences (*p* < 0.05). The figure was created with BioRender.com.

In the SI test (Fig. 6*A*), latency (in seconds) to first approach (Fig. 6*B*) and proximity (Fig. 6*C*) were evaluated. Two-way ANOVA indicated a main effect of Sex (*F*_(1,32)_ = 4.14, *p* = 0.050), but not Treatment (*F*_(1,32)_ = 3.29, *p* = 0.079) or an interaction (*F*_(1,32)_ = 0.75, *p* = 0.39) for latency to first approach. When we analyzed the time spent within a 20 cm proximity, two-way ANOVA indicated a main effect on Sex (*F*_(1,32)_ = 31.62, *p* < 0.0001), with no Treatment effect (*F*_(1,32)_ = 0.86, *p* = 0.36) or interaction (*F*_(1,32)_ = 2.75, = 0.11). Inspection of the data revealed that male offspring approached each other and spent significantly more time within 20 cm of the stranger rat, regardless of treatment.

### Effects of prenatal cannabis smoke exposure on PD/RL

**Figure 7.**
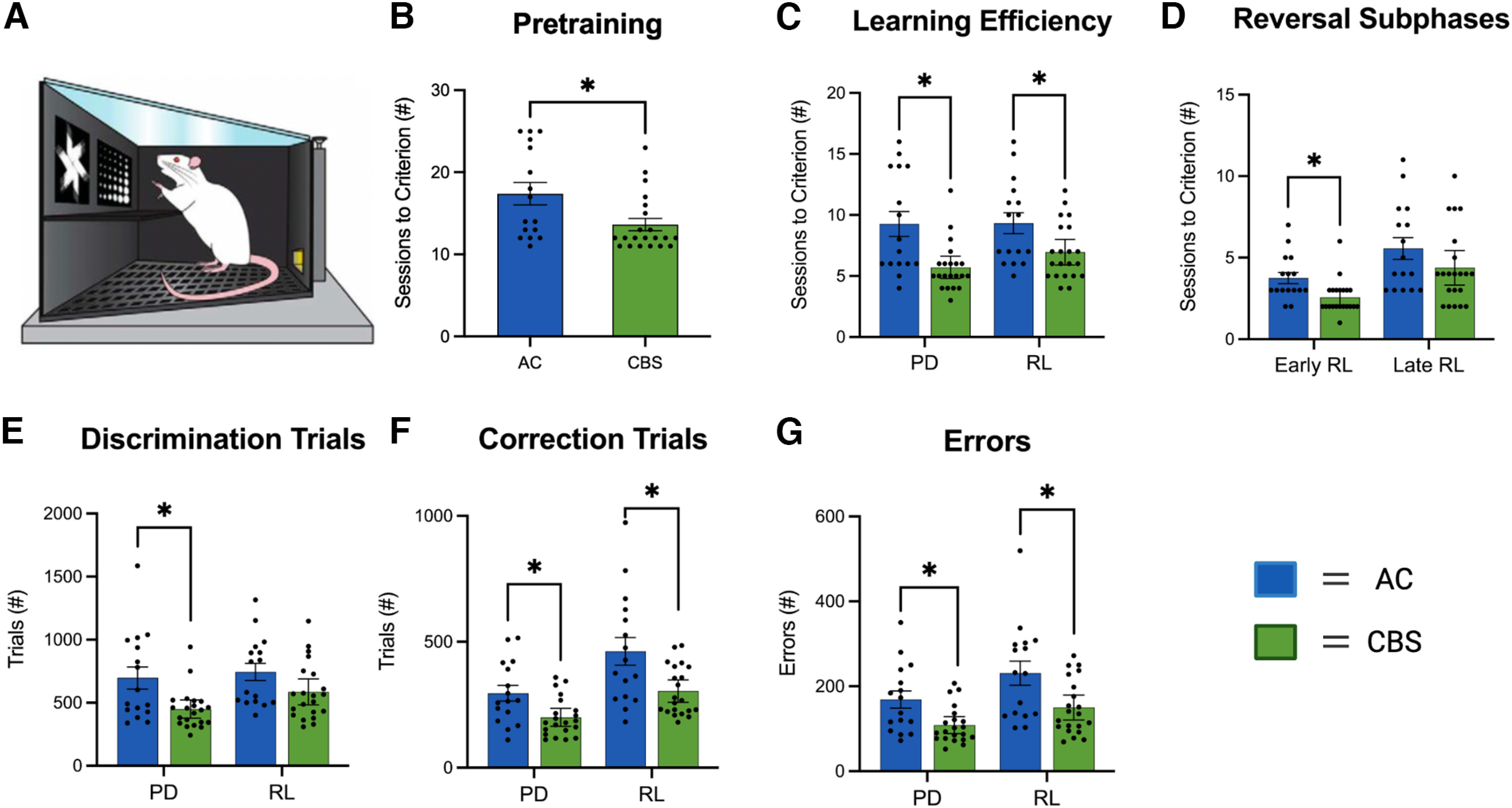
***A***, A separate group of offspring were assessed on visual PD and RL tests in touchscreen-equipped operant conditioning chambers. ***B***, ***C***, Regardless of sex, offspring of high-THC *Cannabis* smoke-exposed dams required significantly fewer sessions for pretraining (***B***) and to acquire the PD and RL tests (***C***). ***D***, *Cannabis* offspring required fewer sessions during the early phase of RL. ***E–G***, Significant differences between groups on discrimination trials (***E***), correction trials (***F***), and errors (***G***) are also noted on the figure where appropriate (see text for further details and discussion). Latencies are summarized in Table 1. Asterisks denote significant changes between treatment groups for each task (*p* < 0.05). The figure was created with BioRender.com.

A separate cohort of male and female offspring were tested for PD and RL (Fig. 7*A*). Regardless of Sex, rats from dams treated with *Cannabis* smoke (*n* = 16) required fewer pretraining sessions for task acquisition than those from control dams (*n* = 21; Fig. 7*B*; Mann–Whitney test, *p* = 0.011). When sessions to complete PD and RL were considered (Fig. 7*C*), analyses revealed that *Cannabis-*treated offspring learned the PD and RL rules in significantly fewer sessions than air controls (Mann–Whitney tests: PD, *p* = 0.002; RL, *p* = 0.02). Analysis of sessions to complete early RL (i.e., all sessions before rats achieve >50% correct on the reversal) and late RL (i.e., all sessions after rats achieve 50% correct on the reversal) revealed a significant difference in early, but not late, RL (Fig. 7*D*; *p* = 0.002, Mann–Whitney test). It is noteworthy that *Cannabis* offspring were also faster than controls during the late RL phase, although results were not significant.

A more detailed analysis of the performance of the offspring during the PD and RL tests revealed subtle differences between treatment groups. When the number of discrimination trials were analyzed (Fig. 7*E*), *Cannabis*-exposed offspring required significantly fewer trials to reach criterion in the PD, but not RL, test (Mann–Whitney, *p* = 0.015). Analyses of correction trials (Fig. 7*F*) and errors (Fig. 7*G*) revealed that offspring exposed to *Cannabis* smoke completed fewer correction trials and made fewer errors than control offspring in both PD and RL (Mann–Whitney test, all *p* values ≤ 0.02). Latencies for correct responses, incorrect responses, and reward collection for PD and RL are shown in Table 1. No significant differences were seen between the treatment groups for any of these measures (Extended Data Table 2-1, statistics).

### Prenatal *Cannabis* exposure altered amygdala gene expression in the adulthood offspring

**Figure 8.**
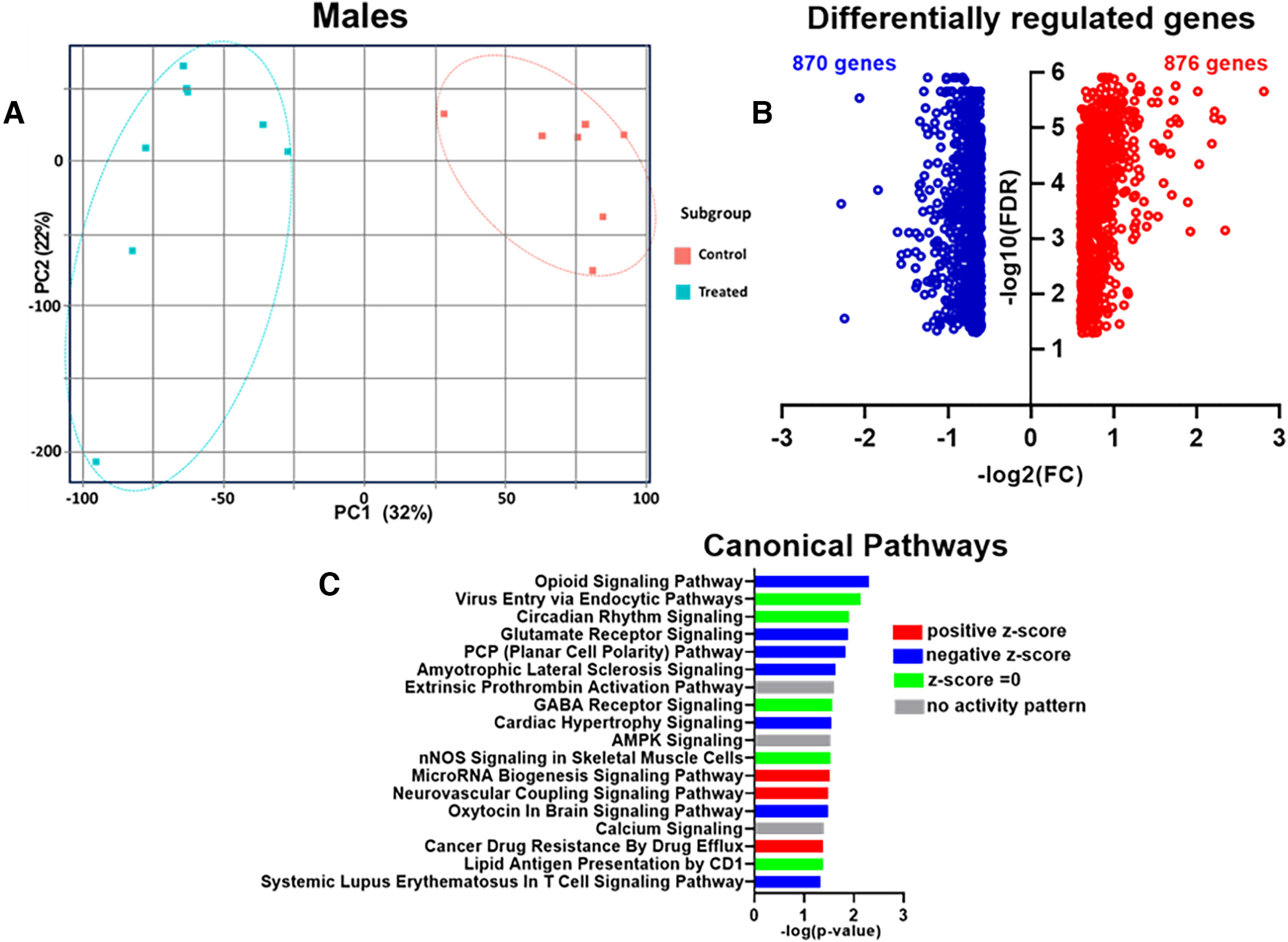
Transcriptional responses in the amygdala of male rat offspring exposed to high-THC *Cannabis* smoke during gestation. ***A***, PCA of RNA-Seq data of male rats. ***B***, Volcano plots display significantly affected genes (FDR < 0.05). Plots of the upregulated (red) and downregulated (blue) genes. ***C***, Significantly enriched canonical pathways. Positive *z* score indicates predicted activation; negative *z* score indicated predicted inhibition. A *z* score of zero indicates no clear direction of activity. No activity pattern indicates insufficient information in the Ingenuity Knowledge Base. Raw data are in Extended Data Table 8-2, *A* and *B*. Additional details related to PCAs and differentially expressed genes in all samples can be found in Extended Data Figures 8-1, 8-2, and 8-3, and Extended Data Table 8-1.

Next, changes in mRNA transcript levels within amygdala tissues of adult rat offspring exposed to *Cannabis* smoke during gestation or control conditions were studied using RNA-Seq. Principal component analysis (PCA) of the expression data showed no obvious clusters corresponding to treatment group within the female samples, and as a result they were excluded from further analysis (Extended Data Fig. 8-1, Extended Data Table 8-1). Furthermore, in the male cohorts, two control male samples and three treated male samples were observed to cluster with samples from the opposite condition and were also excluded from further analyses (Extended Data Fig. 8-2, Extended Data Table 8-1). Transcriptome analysis of the remaining 15 samples revealed a clustering effect in the male offspring exposed to *Cannabis* smoke (*n* = 7) and controls (*n* = 8; Fig. 8*A*). Male offspring exposed to *Cannabis* during gestation had profound transcriptional changes with 1746 differentially regulated genes, of which 876 were upregulated and 870 were downregulated [FDR-adjusted *p* < 0.05; absolute FC > 1.5; Fig. 8*B*, Extended Data Table 8-2*A* (Extended Data Table 8-1, lists of DEGs for all 40 samples and the male and female samples separately]. Among the top 40 affected genes were 23 genes linked to neuronal functions, behavior, or neurodevelopmental disorders (Extended Data Table 8-2, sheet A, GeneIDs).

10.1523/ENEURO.0100-23.2023.f8-1 Figure 8-1 PCA of RNA-Seq data from amygdala samples of all 40 rats. Download Figure 8-1, TIF file.

10.1523/ENEURO.0100-23.2023.f8-2 Figure 8-2 PCA of RNA-Seq data from amygdala samples of the 20 female rats. Download Figure 8-2, TIF file.

10.1523/ENEURO.0100-23.2023.f8-3 Figure 8-3 PCA of RNA-Seq data from amygdala samples of the 20 male rats before outlier correction was applied. Download Figure 8-3, TIF file.

10.1523/ENEURO.0100-23.2023.tab8-1 Table 8-1 DEGs for analyses involving all 40 amygdala samples, 20 female amygdala samples, and 20 male amygdala samples. Download Table 8-1, XLS file.

10.1523/ENEURO.0100-23.2023.tab8-2 Table 8-2 ***A–E***, DEGs (***A***), pathway analysis (***B***), and functional set enrichment analyses (***C–E***) for analyses involving 15 male samples. Download Table 8-2, XLS file.

Accordingly, Ingenuity Pathway Analysis showed significant effects in male offspring exposed to *Cannabis* smoke during gestation with alterations in 18 canonical pathways at a threshold of -log (*p*-value) >1.3 (Fig. 8*C*, Extended Data Table 8-2*B*). Among the top 18 most enriched pathways were 7 pathways involved in neurotransmitters and other nervous system signaling: opioid signaling, circadian rhythm signaling, glutamate receptor signaling, GABA receptor signaling, amyotrophic lateral sclerosis signaling, neurovascular coupling signaling, and oxytocin in brain signaling (Fig. 8*C*).

**Figure 9.**
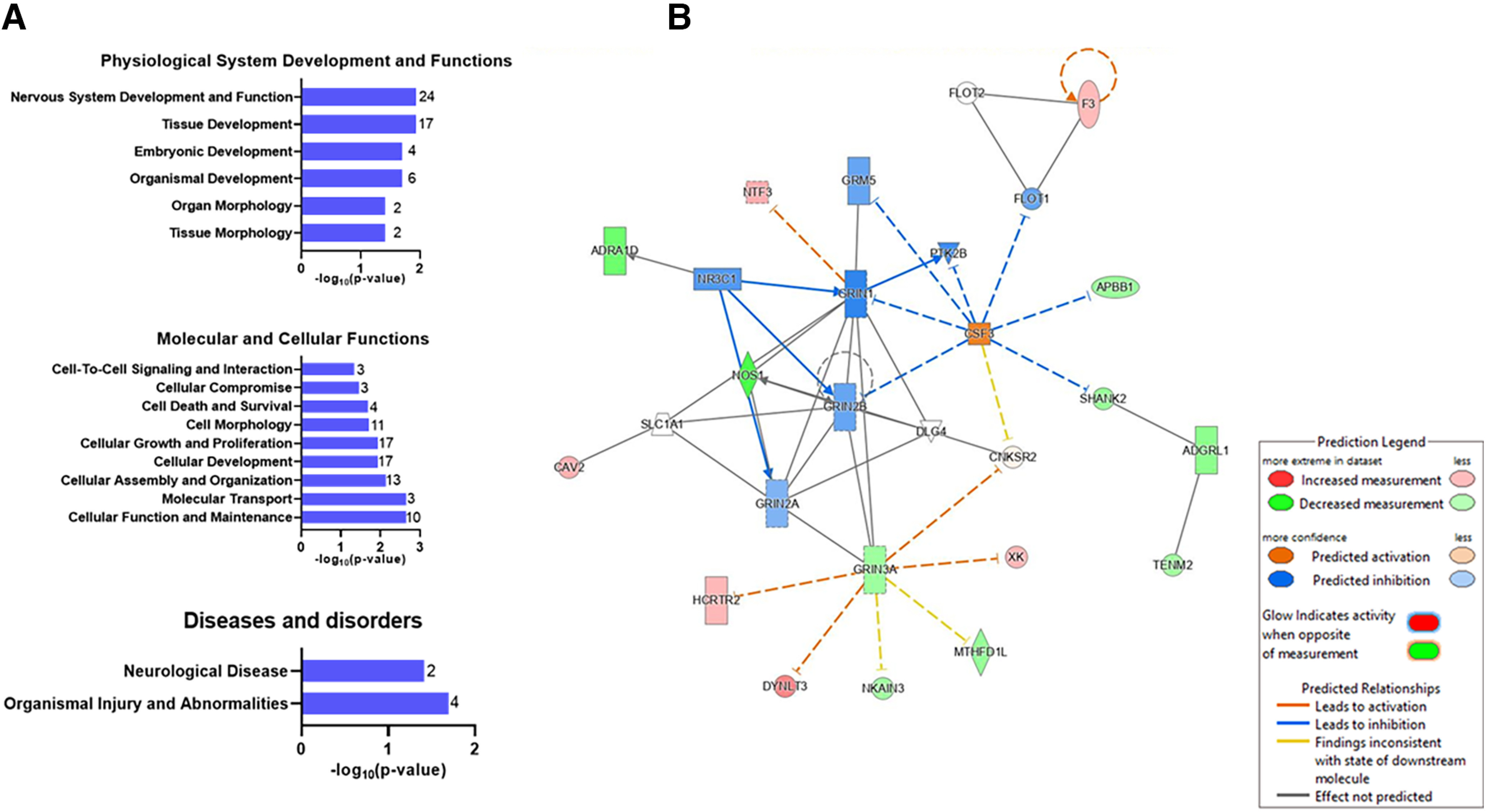
Gene ontology analysis of RNA-Seq analysis from amygdala samples detailed in Figure 8. ***A***, Gene enrichment analysis of differentially expressed genes for biological and disease function identified perturbed pathways in physiological system development and functions, and molecular and cellular disease and disorders. Data are represented as -log_10_ of the lowest *p*-value of an associated pathway. Values at the end of each bar represent the number of differentially expressed genes in each Gene Ontology category. ***B***, Gene interaction network map. This network consists of the top 2 ranked networks of differentially expressed genes linked to neurologic disease, organismal injuries and abnormalities, and psychological disorders. See Extended Data Table 8-2, *C* and *D*, for additional details.

Functional set enrichment analysis for disease and biological processes also showed significant effects in amygdala of male offspring exposed to *Cannabis* during gestation (Fig. 9*A*, Extended Data Table 8-2*C–E*). Specifically, genes with altered expression in the amygdala of exposed rats were predicted to be negatively enriched for processes related to nervous system development, cellular functions, and maintenance and neurologic disease.

Next, we performed network interaction analysis between differentially regulated targets in rats exposed to *Cannabis* during gestation. The top five ranked networks were networks of genes involved in cellular development and functions, nervous system development and functions, neurologic disease, behavior, organismal injuries and abnormalities, and psychological disorders. The top two interaction network maps, which are involved in neurologic disease, organismal injuries and abnormalities, and psychological disorders, have 27 nodes including 15 focus genes from whole-genome transcriptomic targets, as follows: *Adgrl1*, *Adraid1*, *Apbb1*, *Cav2*, *Cnksr2*, *Csf3*, *Dlg4*, *Dynlt3*, *F3*, *Flot1*, *Flot2*, *Grin1*, *Grin2a*, *Grin2b*, *Grin3a*, *Grm5*, *Hcrtr2*, *Mthfd1l*, *Nkain3*, *Nos1*, *Nr3c1*, *Ntf3*, *Ptk2b*, *Shank2*, *Slc1a1*, *Tenm2*, and *Xk* (Fig. 9*B*, Extended Data Table 8-2*F*).

## Discussion

To the best of our knowledge, the present study is the first to examine the effects of commercially available high-THC *Cannabis* smoke administration on rats during pregnancy and their offspring. We show that smoke exposure increases defecation in pregnant rats during the exposure period and significantly reduces their temperature following exposure (Fig. 2). Maternal weight gain and food intake were not altered by the smoke exposure protocol (Fig. 2). Litter number and size were not affected by maternal smoke treatment, although the sex ratio of male to female offspring was significantly increased (Fig. 3). In adulthood, offspring of dams exposed to *Cannabis* smoke during gestation showed evidence of anxiety-like behavior in the OF test (Fig. 4), while treatment effects in the EPM test (Fig. 5) and SI test (Fig. 6) were not observed. Offspring of rats exposed to *Cannabis* during gestation showed facilitated learning of a visual PD and subsequent RL in touchscreen-equipped operant conditioning chambers (Fig. 7). Analyses of amygdala gene expression demonstrated altered expression of genes related to nervous system development and function in a subset of male, but not female, offspring (Figs. 8, 9). Together, these findings suggest that exposure to high-THC *Cannabis* smoke disturbs normal development of the neural circuits involved in emotional and cognitive behaviors.

### High-THC *Cannabis* smoke exposure produced acute changes in physiology of the pregnant dams without dramatic changes in fetal and neonatal parameters

Our maternal *Cannabis* smoke exposure paradigm involved a single daily exposure from GD6 to GD20, which corresponds to the first and second trimesters of human pregnancy. *Cannabis* use is more prevalent during these stages of pregnancy than the third trimester as undesirable symptoms such as nausea are more prevalent (Volkow et al., 2019) and a significant portion of women quit using during pregnancy (Pike et al., 2021). In addition, we did not treat the dams during the earliest stages of pregnancy given the concerns regarding the effects of cannabinoid exposure on implantation and fetal reabsorption (Navarrete et al., 2020), although it is worth noting that some protocols initiate treatments during mating with effects on litter size (Weimar et al., 2020). Daily exposure to high-THC *Cannabis* smoke did not affect the amount of food consumed or maternal weight gain during gestation, thus minimizing concerns related to potential malnutrition on fetal development. However, *Cannabis* smoke exposure significantly decreased rectal temperature, consistent with effects seen in some studies following THC vapor exposure in female rats (Nguyen et al., 2016; Javadi-Paydar et al., 2018), but not others with pregnant rats (Breit et al., 2020). Tolerance to the hypothermic effects of THC vapor exposure have also been noted following twice-daily exposure for 14 d (Nguyen et al., 2020), although we did not observe tolerance in our study perhaps in part because the magnitude of hypothermia produced by our smoke exposure protocol was considerably lower than that described following THC vapor exposure. Quantification of presmoke and postsmoke defecation suggests that our *Cannabis* smoke exposure protocol produced an unintended increase in stress only in the dams exposed to smoke. Interestingly, the number of boli expelled before the *Cannabis* was combusted was reduced after several exposures (Fig. 2*E*), suggesting some degree of habituation to the procedure. However, postsmoke defecation remained high throughout the exposure period (Fig. 2*F*), suggesting that smoke-treated litters were subjected to unintended stress throughout the exposure period. Given the known effects of gestational stress on long-term offspring development in rodent models (Weinstock, 2008), future studies incorporating a placebo smoke exposure protocol using *Cannabis* with THC removed will be valuable for dissociating THC-dependent mechanisms from stress-dependent mechanisms. In addition, measurements of maternal care (Zhang et al., 2012) or cross-fostering of offspring (Weimar et al., 2020) could help mitigate these concerns.

Given the differences in the effects of inhaled vapor or smoke containing THC, analyses of blood levels of THC and its metabolites would help in understanding these differing effects. While we did not measure blood levels of cannabinoids following smoke exposure in this study, acute exposure to the smoke from 300 mg of high-THC containing *Cannabis* results in plasma levels of 10–25 ng/ml after 30 min (Barnard et al., 2022; Black et al., 2023) that drop to <5 ng/ml 75 min after exposure (Roebuck et al., 2022). Extrapolating the plasma levels following exposure to 300 mg of *Cannabis* flower to 200 mg of *Cannabis* flower suggests the present exposure protocol likely produced peak plasma levels that approach the low end of typical blood levels detected in humans (Newmeyer et al., 2016). As dose-dependent effects of perinatal oral THC administration and vaped THC-dominant *Cannabis* have been shown for behaviors related to anxiety, social interaction, and behavioral flexibility (Trezza et al., 2008; Weimar et al., 2020), it will be worth optimizing our smoke administration protocol to achieve higher levels cannabinoid exposure.

Exposure to *Cannabis* smoke using our protocol did not affect litter size or litter weight during the first 2 postnatal weeks. Others have confirmed that inhaled THC exposure during pregnancy in rats does not affect offspring number or weight around the time of parturition (Breit et al., 2020; Baglot et al., 2022), although reduced pup weight has been noted in treated offspring in protocols that exposed rat dams to vaporized THC twice daily during mating and pregnancy (Weimar et al., 2020) and a study of *Cannabis* smoke exposure during gestation in mice (Benevenuto et al., 2017). Thus, both timing of smoke exposure and rodent species could be factors in determining the effects of *Cannabis* smoke on birth weight. In humans, findings related to *Cannabis* use during pregnancy and fetal/neonatal growth are mixed with the Ottawa Prenatal Prospective Study, Generation R Cohort, Avon Longitudinal Study of Parents and Children, and others showing decreased birth weight of offspring exposed to *Cannabis* during gestation, while a number of other studies show no effect of gestational *Cannabis* exposure (for summary, see Grant et al., 2018, their Table 1; Nashed et al., 2021). However, confounds related to relying on self-report of *Cannabis* use, a failure to control for polydrug use, and the increased potency of *Cannabis* products warrant further research in this area (Nashed et al., 2021).

Interestingly, exposure to *Cannabis* smoke significantly increased the male/female sex ratio of the litters. Some previous studies have also reported this effect in mice (Benevenuto et al., 2017) and rats (Hutchings et al., 1987) following exposure to *Cannabis* smoke or orally administered THC. As administration of *Cannabis* smoke was initiated on GD6 in the present study, mechanisms related to the viability of female fetuses following implantation may be more sensitive to the effects of THC or other constituents of *Cannabis* smoke as administered in the present study.

### Offspring exposed to high-THC *Cannabis* smoke during gestation have altered anxiety-like behavior and discrimination learning in adulthood

A subset of adult offspring was tested in the OF, EPM, and SI tests in adulthood to assess locomotor responses, anxiety-like behavior, and social behavior. Total distance traveled in the OF was not altered in *Cannabis* smoke-exposed adult offspring, which is generally consistent with previous studies (Campolongo et al., 2011), and those that show changes in locomotor activity following gestational *Cannabis* exposure only after pharmacological challenge with drugs such as amphetamine (Silva et al., 2012) and THC (Frau et al., 2019). Offspring exposed to *Cannabis* smoke showed reduced distance and time in the inner zone of the OF, measures often used as indices of anxiety-like behavior in rodents. In the EPM, open arm time, a measure related to anxiety-like behavior, did not differ between the treatment groups. While it is unclear why results differed between these two tests, it is noteworthy that several rats in each group failed to enter the open arms in the EPM (Fig. 5*C*, data points with 0 s in open arms). Thus, the room environment for that test may have induced a relatively high baseline level of anxiety in the rats and obscured group differences (i.e., because of a floor effect). Other studies, where open arm exploration was higher in control rats, have revealed an anxiogenic profile in adult rat offspring exposed to THC via oral administration or vapor during gestation (Trezza et al., 2008; Weimar et al., 2020; but see also Bara et al., 2018).

Data from the SI test showed relatively subtle differences between treatments for initial approach latency and proximity that failed to reach significance, particularly for the male offspring. Regardless of treatment, male rats in general spent significantly more time near each other during the test than their female counterparts. Previous studies of the effects of gestational cannabinoid exposure on social interaction in rats are inconsistent with some showing changes in juvenile (Weimar et al., 2020) and adult (Bara et al., 2018) offspring, while others show no effect of gestational cannabinoid exposure on SI in the offspring (Manduca et al., 2020; Traccis et al., 2021). Rodent social behavior is also commonly assayed with the three-chamber “sociability” test (Yang et al., 2011), and gestational exposure to injected THC reduces sociability and social memory in some models (Sarikahya et al., 2023). Therefore, assaying sociability in the offspring of dams treated with *Cannabis* smoke may reveal deficits.

Behavioral flexibility is an executive function essential for optimizing behavioral responses to a dynamic environment and is disrupted in many psychiatric and neurodevelopmental disorders (Hurtubise and Howland, 2017; Soltani and Izquierdo, 2019; Uddin, 2021). Existing preclinical work shows that behavioral flexibility is altered in prenatal exposure models, including maternal immune activation, autism spectrum disorder, and fetal alcohol spectrum disorder (Zhang et al., 2012; Marquardt et al., 2014; Ballendine et al., 2015; Lins et al., 2018; McKinnell et al., 2021). Here, we used a touchscreen-based visual PD and RL paradigm to assess executive function in *Cannabis*-exposed offspring. *Cannabis*-exposed offspring required fewer training sessions to complete PD and RL, suggesting a *Cannabis* smoke-mediated facilitation effect. This facilitation is reflected in several related, but dissociable, performance measures as *Cannabis*-exposed offspring completed these tasks with fewer discrimination trials, correction trials, and errors. Notably, a subset of air control offspring in the present sample performed worse on both PD and RL relative to previous studies, potentially driving the observed facilitation effect (Bryce and Howland, 2015; Lins et al., 2018). In contrast to the present findings, Weimar et al. (2020) found that exposure of rat dams to high-THC *Cannabis* vapor before and during pregnancy marginally increased the number of trials offspring required to reach criterion on the strategy set-shifting component, but not the reversal learning component, of a lever-based task. Hernandez et al. (2021) report that adult rats exposed to *Cannabis* smoke in adolescence exhibit enhanced performance on a delayed response working memory task; however, generalizability of enhancement to the present study is limited by different *Cannabis* smoke exposure timepoints. Ultimately, differences in the specific gestational exposure protocols (frequency and duration of dam exposure) and age of offspring during behavioral testing may account for these observed differences in behavioral outcomes.

### Amygdala gene expression following gestational *Cannabis* exposure

To the best of our knowledge, this is the first time RNA-Seq has been used to assess gene expression in the amygdala of rodents exposed to *Cannabis* during gestation. Analysis of these data demonstrated that *Cannabis* smoke exposure during gestation altered amygdala gene expression in a subset of male offspring during adulthood. As detailed in the Results, more than half of the top 40 affected genes have known functions related to the nervous system. IPA revealed several pathways of interest, including the opioid and glutamate receptor signaling pathways. Changes in these pathways are consistent with previous reports showing altered responses to opioids and glutamate receptors in the offspring of THC-exposed rats and mice (Spano et al., 2007; Frau et al., 2019). Interestingly, increased expression of the preproenkephalin gene (*Penk*) was noted in the central/medial amygdala of adult mice exposed to THC while *in utero* (Spano et al., 2007). While *Penk* levels were not significantly altered in our sample, IPA revealed that genes related to opioid signaling were increased in the amygdala samples from male offspring we collected (Fig. 8*C*, Extended Data Table 2-1*B*).

Some caveats related to interpretation of these data must be considered. First, tissue punches that included the bulk of the amygdala were collected, which precludes analyses of gene expression in discrete amygdala nuclei. Second, this analysis was performed on a subset of male samples as the female samples were not found to cluster when the initial PCA was performed on expression. While sex-related phenotypes have been noted following gestational *Cannabis* exposure, analysis of the present behavioral data did not reveal any sex-by-*Cannabis* exposure interactions in the tasks used. In addition, attempts to relate the gene expression patterns with individual offspring anxiety-like behavior in the OFT failed to account for the lack of consistently clustering in the female samples (data not shown). Given the role of the amygdala in behavioral flexibility, and reversal learning in particular, the gene networks altered in the offspring may have also contributed to the facilitated performance we observed on this task. However, as we failed to observe a sex difference here too, firm conclusions cannot be drawn. Third, while it is tempting to attribute the changes in gene expression to an enduring, direct effect of *Cannabis* exposure while the offspring were *in utero*, they may instead reflect compensatory changes in the amygdala from other unknown effects of *Cannabis* exposure on offspring development and behavior, including the OF, EPM, and SI tests these offspring were exposed to before the amygdala samples were taken.

Previous research has assessed patterns of gene expression in other brain regions following gestational *Cannabis* exposure (Scheyer et al., 2019; Bara et al., 2021). In particular, Campolongo et al. (2007) found that 141 genes were differentially expressed in the prefrontal cortex of male offspring of dams administered oral THC from GD15 to P9. Networks of genes related to synaptic transmission, development, neurogenesis, and myelination were altered, including several genes (*Grik3*, *Neurod2*, *Rtn4*, *Mobp*) that were also altered in the amygdala samples assayed in the present study. Reduced *Drd2* (DiNieri et al., 2011) and increased *Penk* mRNA levels (Spano et al., 2007) have been observed in the nucleus accumbens of rats exposed to injected THC during gestation. Others have not found differences in vesicular transporters for glutamate, GABA, and acetylcholine or SNARE proteins in cortex of the adult offspring of mice exposed to injected THC during gestation (Tortoriello et al., 2014).

### Conclusion

Together, our results suggest that the repeated exposure of pregnant rat dams to high-THC *Cannabis* smoke alters some behaviors related to anxiety and behavioral flexibility in adulthood. These behavioral alterations may relate to disturbed patterns of amygdala gene expression that are also expressed in adulthood. Future research to assess the effects of other *Cannabis* products, such as high-CBD products, changes in transcript abundance in other brain regions, and other behavioral outcomes of maternal *Cannabis* exposure are now underway.

## Synthesis

Reviewing Editor: Mark Laubach, American University Decisions are customarily a result of the Reviewing Editor and the peer reviewers coming together and discussing their recommendations until a consensus is reached. When revisions are invited, a fact-based synthesis statement explaining their decision and outlining what is needed to prepare a revision will be listed below. The following reviewer(s) agreed to reveal their identity: Ryan McLaughlin, Jibran Khokhar.

Your manuscript was reviewed by two experts and was favorably scored. Some revisions are needed before a decision can be made on your manuscript. The comments of the reviewers are below. Please revise your manuscript to address all points that were raised. Thank you for sending your work to eNeuro.

Reviewer #1

This manuscript examines effects of maternal cannabis smoke exposure on offspring development, exploratory behavior, cognitive flexibility, and amygdala gene expression. The authors find effects of prenatal cannabis exposure on several endpoints, including sex ratio (male>female), open field exploration (less center quadrant exploration in cannabis offspring), pairwise discrimination and reversal learning (cannabis offspring unexpectedly performed better than air controls). Additionally, RNAseq and Differential Gene Expression analyses on amygdala tissue collected postmortem revealed 1746 differentially regulated genes in male offspring (female samples were unable to be analyzed), a few of which appear to be consistent with published literature, namely opioid and glutamate receptor signaling pathways. Overall, this is a solid contribution to the field that adds to a growing body of literature that have used preclinical approaches that use whole-plant cannabis preparations to model more accurately the most common method of cannabis consumption in humans (i.e., smoke inhalation). The paper is generally well written, the statistical approaches are appropriate, and the authors do a reasonable job of providing explanations for discordant or unexpected results in the discussion. I just have a few minor to moderate comments that I hope the authors can address, as I feel that it will only further strengthen an already strong manuscript.

Can the authors clarify how many pups/sex from each litter were used for each experiment? Litter effects are important to consider, particularly if pups from one litter are skewing the data. If more than 1-2 pups/sex/treatment group were used from each litter for a given experiment, this should be noted.

It should be noted in the discussion that unintended effects of stress due to forced smoke exposure could have also influenced the various endpoints measured, particularly when it comes to stress-related behaviors (open field center exploration), and stress-sensitive brain regions (i.e., the amygdala). The authors do a good job of habituating all animals to the smoke delivery system prior to initiating the experiment, but it is concerning that the smoke-exposed group had significantly more fecal boli, which suggests a higher degree of stress in this group (to my knowledge, cannabis is not particularly effective at stimulating bowel movements in humans). This is clearly impossible in retrospect, but a clear limitation of these data is the lack of a “placebo cannabis” group that is exposed to hemp-derived flower that lacks THC. This is what is typically used in human studies exploring acute effects of cannabis in the lab. I suggest that this be noted in the discussion.

Could the authors clarify why they chose to conduct the social interaction test in this manner? Typically, one of the rats is restrained under a mesh device so that exploration of the novel conspecific can be measured independent of the activity/social preference of the conspecific. This approach here seems like it would be challenging to score and thus difficult to properly interpret.

I am much less familiar with RNAseq approaches, but it seems very concerning that the PCA showed no clusters by treatment group in females, and that some male samples from each group seemed to cluster in the opposite direction (and were thus simply excluded). This seems problematic. Could the authors elaborate briefly on why they might have received these unexpected results? To me, I’m not sure that the RNAseq data in its current form really adds very much to the manuscript as it seems to be reviewed and discussed somewhat superficially relative to the behavioral endpoints.

Minor point:

In the discussion, the authors incorrectly refer to the behavioral flexibility deficit shown from Weimar et al., 2020 as a “marginal increase” in trials required to complete “a visual cue discrimination task”. The effect in this paper was specifically in the extradimensional set-shifting component of the task, not the visual cue discrimination component. In fact, in that study some of the low dose exposure animals (50 mg/ml THC) were among the best responders for visual cue discrimination, which is consistent with these data. Also, the authors misspell “Weimar” at the bottom of Page 23.

Reviewer #2

This paper aims to understand the effects of cannabis smoke exposure during gestational days 6-20 in female Sprague-Dawley rats. Physiological changes in the dams (including body temperature and number of fecal boli) were observed after cannabis exposure, whereas maternal weight gain, food intake and gestational parameters (such as length, litter size and weight) were not affected. However, there was an increase in male to female pup ratio in the cannabis exposed litters. Both male and female pups exposed to cannabis smoke during gestation showed lower exploration in an open field but no differences in elevated plus maze or social interaction. Cannabis exposed offspring were better at touch-screen based pairwise discrimination and reversal learning. The male offspring also showed changes in gene expression. This is an important study that could be improved via some suggestions below:

Major changes:

Introduction:

Page 3 Line 7: Variable in what ways? What proportion is using cannabis daily among those who report cannabis use in pregnancy?

Justification for high THC specifically not addressed. Are pregnant individuals who smoke cannabis seeking high THC content, or just exposed to it by virtue of increasing concentrations in THC content?

What is the justification for looking at the amygdala exclusively?

Methods:

Breeding: If rats are confirmed pregnant by G0, why initiate exposure at G6? Presumably a portion of pregnant individuals will consume cannabis in the earliest stages of embryo exposure before knowing they are pregnant.

Was the THC content from the earlier papers (which were used to justify amount) similar to the strain used here?

“Significant exposure occurred during this time” How long was residual smoke left in the chamber?

Did the control air procedure include turning pumps off for the 1min period when smoke exposure would have occurred?

Unclear exactly when boli are recorded. In the room transportation equipment? In the chamber during the 5 min before smoke exposure and ∼10 min after exposure?

Open field: How is the center defined? Latency to enter open arm? Why of the same treatment?

Social Interaction: If THC influences SI, there could be a compounding effect if both animals were affected by exposure.

PD/RL: Unclear what a “response self” is?

What are the primary measures of interest for PD and RL that lead to exclusion?

Why was gene expression only assessed in the OF, EPM and SI group? Assessing in both groups would help to ensure there was no treatment by behaviour interaction on gene expression.

Results

The use of “Time” as a parameter here may cause confusion. Is time the pre-post distinction, or is it the time over gestation (i.e., day)? Was there any day effect? Did the cannabis exposed dams show tolerance development?

A change in body temp may be more useful than individual analysis of before/after temperature recordings.

Gene analysis: Is a combined sex analysis possible? Does that reveal any unique clustering?

With already having a small n, excluding 5 animals could greatly affect the data. How does the data look if these outliers are retained?

For the list of 23 genes: Why list all of these here? If you must include them, alongside what is presumably a reference to their neuronal function/behavior/neurodevelopmental disorder, it would be worth mentioning what the specific association is.

Discussion:

A possible explanation for any other treatment differences is that the cannabis smoke could be inherently more stressful to the dams regardless of any THC content. There is a lot of literature on stress and gestation, and so this alternative should be considered and addressed as a major limitation.

Acute changes in physiology section:

More prevalent than what? Pre-pregnancy? The third trimester? Statistics in the introduction about the course/rates of cannabis use over these times would be helpful

Mixed findings in humans: This is vague and should be expanded upon. Are there an equal number of publications support no vs reduced (vs increased?) infant weight? Or does support lean one way or the other? What animal models might be better in light of these equivocal human findings? Are there any human complement studies around sex ratios born to those who use vs don’t use cannabis in pregnancy?

The fact that many rats failed to enter the open arms in the EPM is important but was not mentioned in the results section. How many of each group?

Is there any evidence of maternal care differences in the cannabis treated vs non-treated dams? Litter hierarchal patterns? How could this be addressed if not? Is there any literature to support this potential confound?

If these other reports of changes in PFC exist, what is the justification for just looking at the amygdala?

Figures:

Figure 2 body weight and rectal temperature: Why show overall weight gain and not weight gain over time? Same as weight gain, it would be informative to include body temperature measurements over the days.

Minor changes:

Introduction:

Some grammar related issues (e.g., is crucial for a better understanding).

The full species name should be written in full at the beginning if you plan to use the species italics notation for the full manuscript.

Some of the rationale for the study (E.g., developmental changes etc) are vague. In what domains were these changes observed specifically? What are the most pressing research concerns that remain unaddressed? Are they expecting the same thing as injection? If so, why is this important to investigate?

Some redundancy: Justification for high THC specifically not addressed. Are pregnant individuals who smoke cannabis seeking high THC content, or just exposed to it by virtue of increasing concentrations in THC content?

Results: Would be good to stay consistent with significant figures.

Page 17, line 2: Confirm you mean faster to complete the session, not faster by some locomotor measure

Discussion:

Page 20, Line 14: Would be useful to state this in the methods. Citation for this?

Was tolerance to hypothermia seen in this study?

More specific language would be helpful (e.g., altered).

Several times through the manuscript, other studies are referred to with “exposed to THC”, etc. A key thing to include with these statements is how the route of exposure.
