# Supplementary figures and images for: Repeated Exposure to High-THC Cannabis Smoke during Gestation Alters Sex Ratio, Behavior, and Amygdala Gene Expression of Sprague Dawley Rat Offspring
Source: eNeuro. 2023 Nov 27;10(11):ENEURO.0100-23.2023. doi: 10.1523/ENEURO.0100-23.2023 (PMC10687874; doi:10.1523/ENEURO.0100-23.2023)

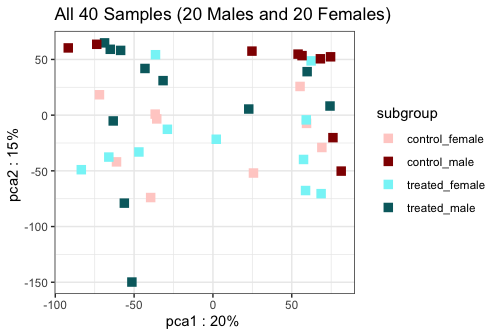

Supplement: Figure 8-1 — PCA of RNA-Seq data from amygdala samples of all 40 rats. Download Figure 8-1, TIF file. [file enu-eN-NWR-0100-23-s02.tif]

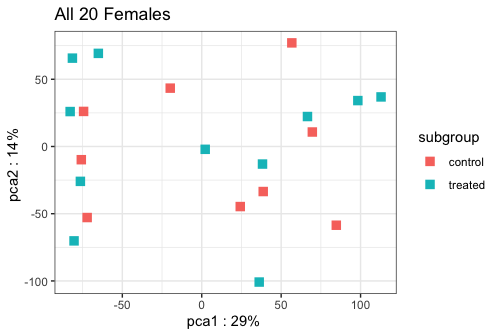

Supplement: Figure 8-2 — PCA of RNA-Seq data from amygdala samples of the 20 female rats. Download Figure 8-2, TIF file. [file enu-eN-NWR-0100-23-s03.tif]

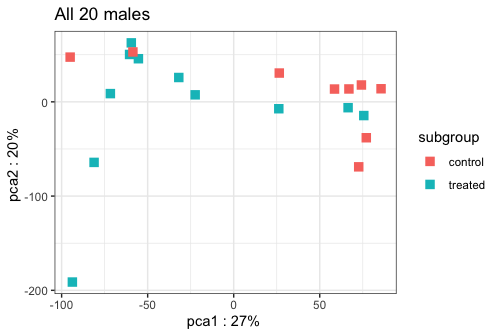

Supplement: Figure 8-3 — PCA of RNA-Seq data from amygdala samples of the 20 male rats before outlier correction was applied. Download Figure 8-3, TIF file. [file enu-eN-NWR-0100-23-s04.tif]
